# Elements of Anatomy

**Published:** 1846-04

**Authors:** 


Elements of Anatomy. By *Jones Quain*, M.D. Edited by Professors *Richard Quain* and *William Sharpey*, M.D. 8vo. Part 2. Taylor and Walton, 1846.

A period of more than two years has intervened between the appearance of the first and second parts of Dr. Quain's useful work ; a delay which might have been avoided if the able editors had infused a little more activity into their proceedings. We have thought it right to notice this circumstance, because the patience of purchasers has, of late years, been considerably tried in matters of this kind. The principal feature of this new edition, is the introduction of a concise account of the structural or general anatomy of the various fluids and tissues, from the pen of Dr. Sharpey. This additional matter occupies in the first part eighty-four, and in the present volume one hundred pages.

There is a process constantly proceeding in the animal economy, which, although it must be classed among the most important of the vital actions, is but very imperfectly understood ; we allude to the production or formation of the red particles of the blood. The vast activity with which the metamorphosis of the various organs is incessantly proceeding and the consequent conversion of the blood, notwithstanding the fixed attention that has been latterly directed to that process, owing chiefly to the brilliant expositions contained in the writings of Liebig, is scarcely yet duly estimated by physiologists. It is apparent that the nearest approximation to the measure of these changes would be obtained by determining the actual amount of matter poured into the blood in a given time by the thoracic duct and by the right trunk of the lymphatic system, as those are the channels by which all the débris of the system and all the new nutritive matter are conveyed. According to a series of experiments lately performed by Professor Bidder of the University of Dorpat, and recorded in Müller's Archiv. for the last year, the quantity of fluids passing through the thoracic duct alone in 24 hours is, in cats, equal to the amount of the whole of the blood contained in the body ; in dogs, it is about one-third less. Even granting that these estimates, which exceed those of other authors, are exaggerated, it is certain that canals of the large size of those constituting the two lymphatic trunks, constantly pouring their contents into the venous system, must introduce a large amount of fluid per diem ; and further, admitting, what is proved by various observations, that the red corpuscles are not changed and renewed with the same facility as the other constituents of the blood, still the reproduction of these bodies must be incessantly proceeding.

As to the mode in which this reproduction is effected, the most conflicting accounts have been given : on one point, however, all observers are agreed, that whatever may be the future form of the particle, whether circular and bi-concave, as in mammals, or oval and bi-convex, as in birds, reptiles and fishes, it commences as a round vesicle or cell. In reference to the process in the blastoderma or germinal membrane of the ovum, Dr. Sharpey observes, " as to the earlier part of the process,—the production of the above-mentioned round cells, whose subsequent conversion into coloured oval discs has just been described,—the statements of observers differ so widely, that no consistent account can be founded on them. By one it has been imagined, that they are formed from the oil globules known to exist in the yolk, which serve as nuclei, and become enclosed in envelopes. Reichert supposes that they are produced within parent cells, which proceed from the central part of the yolk to the germinal membrane, generate round nucleated blood-corpuscles in their interior, and discharge them, by rupture, into the blood-vessels. Another inquirer finds, that the blood-corpuscle begins as a small granule, which rapidly enlarges into a spherical cell, and separates into nucleus and envelope. Lastly, Prevost and Lebert declare, that the blood-corpuscles, even on their first appearance, are round, slightly flattened, colourless, nucleated cells, which differ from all other cells in the ovum; and they could find no transitional forms indicating a transformation of any of the pre-existing cells of the ovum into these early blood-cells." P. xci.

With respect to the embryo of mammiferous animals the author adopts the views of Wagner and Bischoff, that the primary blood-cells are round, nucleated, and colourless cells, not differing in appearance from the common primary cells of which all the solid parts of the embryo are at first composed. Subsequent to birth the new red particles are, with other physiologists, supposed to be derived from the corpuscles of the lymph and chyle, which, passing into the sanguiferous system, become the pale corpuscles of the blood, whilst these, in their turn, becoming flattened, losing their nuclei, and acquiring colouring matter, are thus gradually converted into red discs: the writer, however, further thinks, that the pale or colourless corpuscles may be also generated in the blood-vessels themselves.

One of the interesting results of microscopic anatomy is the establishment of the fact, that the skin of the dark races of mankind does not contain any peculiar layer, the so-called rete mucosum, which is wanting in the Caucasian variety.

"The dark colour of the Negro is known to have its seat in the cuticle, and chiefly in the deeper and softer part named the rete mucosum. According to Henlé, it is caused by the presence of pigment cells, resembling those of the choroid in almost every respect save their size, which is somewhat less. These are intermixed with colourless cells, and on the proportion of the two the depth of colour of different parts depends. According to the same authority, the darker parts of the European skin owe their colour to pigment cells like those of the Negro, only still smaller in size, less defined in their outline, and less numerous." P. cvii.

The mechanical texture of bone, when viewed in a collective manner, offers a beautiful example of the doctrine of final causes in the animal organism; in fact, the most minute parts revealed by the microscope present only a repetition of what has so long commanded the admiration of all reflecting minds when seen by the naked eye. The Haversian canals—the lacunæ, nay, even the canaliculi—what are these but the central hollow cylinder, and the cells of the cancelli, on an extremely small scale? Anatomists, indeed, are content with regarding them as being essentially passages for the conveyance of the nutritious fluids, but we hold the primary object of the whole formation to be *mechanical*, affording, with the least possible amount of material, the greatest quantum of resistance.

The section on this subject, and on the formation of bone, to which it is well known Dr. Sharpey has contributed many important facts, is very complete, and will repay a careful perusal. A minute account is given of the lamellæ surrounding, in a series of concentric rings, the Haversian canals. When examined in a bone deprived of its earthy matter, the lamellæ are seen to be perforated with fine apertures, described originally by Deutsch, and which Dr. Sharpey conceives to be nothing else than the transverse sections of the minute pores, known as the canaliculi.

"According to this view, therefore, the canaliculi might (in a certain sense) be conceived to result from the apposition of a series of perforated plates, the apertures of each plate corresponding to those of the plates contiguous with it; in short, they might be compared to holes bored to some depth in a straight or crooked direction through the leaves of a book, in which case it is plain that the perforations of the adjoining leaves would correspond.

"But the lamellæ have a further structure. To see this the thinnest part of a detached shred or film must be examined; it will then appear plainly that they are made up of transparent fibres, decussating each other in form of an exceedingly fine net-work, and that the perforations correspond to the intervals or openings between the reticulated fibres. The fibres intersect obliquely, and they seem to coalesce at the points of intersection, for they cannot be teased out from one another; but at the torn edge of the lamellæ they may often be seen separate for a little way, standing out like the threads of a fringe. Most generally they are straight, as represented in the figure; but they are not always so, for in some parts they assume a curvilinear direction. Acetic acid causes these fibres to swell up and become indistinct, like the white fibres of cellular and fibrous tissue." P. cxliii.

As to the ossific process, the author confirms the opinion that, although bones generally are formed in a nidus of cartilage, yet, that the tabular bones of the skull are developed in a membranous tissue of a totally distinct nature. In this, the intra-membranous form of ossification, "the growing bone shoots into the soft tissue, in form of transparent fibres, resembling those of fibrous texture, more or less intermixed with granular corpuscles, and these fibres become charged with earthy salts;" the cells are also involved in the process, but in what way is not apparent. Another instance of this *intra-membranous* ossification is afforded in the deposit of bone which takes place immediately beneath the periosteum, in which situation the vascular soft tissue in direct contact with the surface of the growing bone is not cartilage, but consists of fibres and granular corpuscles; "in fact, the increase takes place by intra-membranous ossification, and accordingly the Haversian canals of the shaft are formed in the same way as those of the tabular bones of the skull." Some novel details are recorded relating to the process as it occurs in cartilage, which we regret our limits will not allow us to extract.

In the account of the muscular tissue a very interesting description and representation are given of some preparations made by Mr. Lealand, a skilful optician residing in the metropolis.

"When a fibril thus completely insulated is highly magnified, it is seen to consist of a single row of minute particles, connected together like a string of beads. These particles (named 'sarcous elements' by Bowman), when viewed with a magnifying power of 400 or 600, appear like little dark quadrangular and generally rectangular bodies, with bright intervals between them, as if they were connected together by some pellucid substance; but on closer examination, provided the defining power of the instrument is good, a very faint dark line or shadow will be discovered passing across the fibril in the middle of each of the bright spaces, and sometimes also a bright border may be perceived on either side of the fibril, so that each of the rectangular dark bodies appears then to be surrounded with a bright area having a similar quadrangular outline, as represented in the figure, and it may therefore be inferred that the pellucid substance incloses it on all sides. In short, it would seem that the elementary particles of which the fibril is made up, are little masses of pellucid substance, presenting a rectangular outline, and appearing dark in the centre. Their appearance, indeed, suggests the notion of minute vesicular bodies or cells, cohering in a linear series, the faint transverse marks between being the lines of junction. But although this idea very naturally presents itself, we must not assume that the reality of it is established. With a still higher magnifying power, the dark central part appears constricted in the middle, or looks as if it consisted of two portions joined together. When the focus is altered, the internal dark part becomes light; it is therefore evidently transparent, and its dark aspect is probably owing to its refracting the light differently from the surrounding substance." P. clxviii.

This additional and indisputable proof of the quadrangular outline of the sarcous elements, must remove any doubts, if such still exist, as to entire accuracy of Mr. Bowman's observations.

We have not space to make any special reference to the descriptive and surgical anatomy of the vascular and nervous systems, edited by Mr. Quain. It would, however, be unjust to close this article without stating that very considerable additions have been made in this division of the work, advantage having been taken of the numerous contributions by which these branches of anatomical science have been enriched of late years, both in this country and on the Continent. These "Elements" may, on the whole, be regarded as constituting one of the most valuable works to which the medical student can apply for assistance in the prosecution of his studies; and we may further safely recommend them to those who are engaged in the more active duties of the profession, on the ground of the judicious combination herein effected of sound scientific research with instructive practical details.

